# Investigating the mobility and host range of mobile genetic elements harbouring antimicrobial resistance genes in enterococci

**DOI:** 10.1099/mic.0.001720

**Published:** 2026-07-02

**Authors:** Jee In Kim, Rahat Zaheer, Athanasios Zovoilis, Gary Van Domselaar, Sani-e-Zehra Zaidi, Travis Haight, Robert G. Beiko, Tim A. McAllister

**Affiliations:** 1 Faculty of Computer Science, Dalhousie University, Halifax, Canada; 2 Institute for Comparative Genomics, Dalhousie University, Halifax, Canada; 3 Lethbridge Research and Development Centre, Agriculture and Agri-Food Canada, Lethbridge, Canada; 4 Department of Biochemistry and Medical Genetics, University of Manitoba, Winnipeg, Canada; 5 National Microbiology Laboratory, Public Health Agency of Canada, Winnipeg, Canada

**Keywords:** antimicrobial resistance, *Enterococcus faecalis*, *Enterococcus faecium*, *Enterococcus hirae*, horizontal gene transfer, mobile genetic elements

## Abstract

In this study, the abundance and conjugation capacity of mobile genetic elements (MGEs) carrying resistance genes such as *vanA*, *tet*(M) and *erm*(B) were investigated to enhance our understanding of antimicrobial resistance (AMR) dissemination across the One Health continuum in high-priority, highly prevalent enterococcal pathogens. The abundance of MGEs was estimated using replicon typing and both reference-based and reference-free clustering approaches. Conjugation potential was assessed using agar plate mating between *Enterococcus faecium* donors and *E. faecium*, *Enterococcus faecalis* and *Enterococcus hirae* recipients, with conjugated MGE verified via long-read sequencing. Key findings include the identification of a *vanA* gene cluster from *E. faecium* VRE0008 associated with a Tn*1546*-like transposon embedded in a RepA_N-type putative plasmid (232,902 bp). This plasmid successfully conjugated with *E. faecium*, *E. faecalis* and *E. hirae* recipients from clinical, environmental and agricultural sources. The transfer predominantly involved the modular movement of a 46-kb region surrounding the *vanA* gene cluster, with *E. hirae* of agricultural origin (i.e. 0093A) being the exception, as it retained the entire plasmid. The *tet*(M) gene from *E. faecium* Ent0189 was located on a putative Rep_Trans-like plasmid, with features of Tn*916* integrative conjugative elements. The entire plasmid from Ent0189 was successfully transferred to intra-species recipients from clinical and environmental sources, but transfer to *E. faecalis* and *E. hirae* was less common. Attempts to transfer *tet*(M) associated with Tn*916* from bovine *E. hirae* to any of the *E. hirae*, *E. faecium* and *E. faecalis* isolates were unsuccessful. Additionally, the *erm*(B) gene from *E. faecium* NS0794 was carried by an MGE matching the RepA_N-type plasmid, but lacking the *vanA* gene cluster. Successful conjugative transfer of this plasmid was observed with *E. faecium*, *E. faecalis* and *E. hirae* of various origins, except one clinical *E. faecalis* isolate. These findings highlight the broad conjugation capabilities and modular mobility of MGEs carrying ARGs in enterococci, enhancing our understanding of dynamic MGE-mediated ARG dissemination and informing strategies to address the spread of AMR between species and habitats.

## Data Availability

The donor and recipient strains were obtained from BioProject PRJNA604849 [1], with corresponding accession numbers listed in Table 1. Long-read draft genome assemblies of *Enterococcus* spp. transconjugants have been deposited in GenBank under BioProject PRJNA1358929 (BioSamples SAMN53131007, SAMN53131008, SAMN53131009, SAMN53131010, SAMN53131011, SAMN53131012, SAMN53131013 and SAMN53131014). One transconjugant assembly (pVRE0008+*Enterococcus faecium* ATCC 51558) did not meet the minimum genome size requirements for submission to the National Center for Biotechnology Information (NCBI) assembled genome database after contaminating sequences were removed. Nevertheless, the remaining assembly retained sufficient genomic context to confirm successful conjugative transfer and to identify the transferred genetic element.

**Table 1.** (A) Donor and (B) recipient *Enterococcus* spp. strains used in the conjugation experiment

A. Donor strain	Origin	Description	Accession
*E. faecium* VRE0008	clinical	contains *vanA* gene cluster and *erm*(B)	SRR14010950
*E. faecium* Ent0189	natural water sources	contains *tet*(M)	SRR14026472
*E. faecium* NS0794	clinical	contains *erm*(B)	SRR14026517
*E. hirae* ES-A-FC103–20 OCT14-0200A	bovine faeces	contains *tet*(M)	SRR19576962
**B. Recipient strain**	**Origin**	**Description**	**Accession**
*E. faecium* ATCC 51558	ATCC strain	*	ATCC Genome Portal
*E. faecium* NS1659	clinical	*	SRR14026519
*E. faecium* ES-M-ST001-03MAR15-0067A	urban wastewater	*	SRR14022776
*E. faecalis* ATCC 47077	ATCC strain	*	CP002621.1
*E. faecalis* ES-C-ST001-18APR16-0145C	urban wastewater	*	SRR13725728
*E. faecalis* NS0672	clinical	*	SRR13999985
*E. faecalis* BP R37-3	retail beef	*	SRR13712366
*E. hirae* ATCC 9790	ATCC strain	*	CP003504.1
*E. hirae* ES-V-CB001-26MAY15-0093A	feedlot catchbasin	*	SRR19576993
*E. hirae* ES-A-SW-14APR14-Ent0007	natural water sources	*	SRR19576906
*E. hirae* ES-M-ST001-23NOV14-0060A	urban wastewater	*	SRR19577147

* Recipient strains were experimentally adapted to develop rifampicin and fusidic acid resistance.

## Introduction

Enterococci are ubiquitous Gram-positive bacteria found in soil, plants, water and the intestinal tracts of humans and animals [2,5]. Although they can reside as gut commensals, certain species, such as *Enterococcus faecium* and *Enterococcus faecalis*, have emerged as some of the leading causes of hospital-acquired infections in humans over the past few decades due to intrinsic and acquired resistance [6,7]. Their intrinsic resistance to a wide range of antimicrobial drugs, including cephalosporins, aminoglycosides and trimethoprim-sulfamethoxazole, limits treatment options, complicates therapy decisions and increases the risk of treatment failure [8]. In addition, the rising incidence of acquired resistance in enterococci further amplifies these concerns, highlighting the clinical relevance of these opportunistic pathogens. Since the inception of the Bacterial Priority Pathogens List in 2017, the World Health Organization (WHO) has included antibiotic-resistant *E. faecium* as a target to prevent and mitigate the spread of antimicrobial resistance (AMR) among the most dangerous antibiotic-resistant bacteria [9].

Enterococci genomes exhibit remarkable plasticity, enabling them to acquire antimicrobial resistance genes (ARGs) through horizontal gene transfer (HGT) that confer resistance to numerous antibiotics, including ampicillin, vancomycin, tetracycline, gentamicin and linezolid [10, 11]. The genomes of multidrug-resistant strains are typically at least 20% larger than commensals as they commonly lack CRISPR-Cas loci, which promotes the accumulation of mobile genetic element (MGE)-like phages, plasmids, pathogenicity islands, integrative and conjugative elements (ICEs) and transposons that carry ARGs [12,17]. Conjugation, the process of transferring plasmid DNA from a donor to a recipient bacterium via a specialized multiprotein complex, is one of the most common HGT mechanisms in enterococci [15,18]. The ability to share genes has not only enabled enterococci to evolve and persist in various environments [19,20] but also makes them excellent reservoirs of MGEs, which contribute to the spread of AMR and virulence genes [21,23]. Understanding how well enterococci share genes across different environments will help establish appropriate preventive measures.

Our previous study employed machine learning (ML) to predict genomic features associated with resistance to vancomycin, doxycycline and erythromycin in 647 *E. faecium* and *E. faecalis* isolates [24]. As expected, ARGs were the most significant predictors of resistance, with a strong linkage to MGEs. The ARGs were specifically associated with transposable elements (TEs) and conjugative plasmids, suggesting that HGT plays an important role in the ecology of AMR in enterococci. Building on these insights, the present study aimed to investigate the dissemination capacity of MGE-associated ARGs in *Enterococcus* species guided by the predictors from our previous genomic analysis. We characterized the structures of representative MGEs and assessed their putative abundance in our dataset. Conjugation capacity was tested using *Enterococcus* recipients of various origins, and the transferred MGEs carrying ARGs were confirmed and identified through sequencing.

## Methods

### Genome datasets and annotation

A total of 647 sequenced enterococci isolates from clinical, agricultural, municipal wastewater, agricultural wastewater and natural water sources of Alberta, Canada, with corresponding categorical resistant/susceptible phenotypes were used in this study (BioProject PRJNA604849) [1,24]. Of these, 517 genomes (237 *E. faecium* and 280 *E. faecalis*) were short-read assemblies, while 131 genomes (73 *E. faecium* and *58 E. faecalis*) were hybrid short- and long-read sequences originating from Illumina and PromethION (Oxford Nanopore Technology), respectively. The short-read assemblies were completed using the ARETE pipeline (https://github.com/beikolab/arete) as described previously [24]. Long-read sequences were assembled with Flye (v2.9.3; with parameter ‘–asm-coverage 50’), and the assembled contigs were used along with short-read sequences to generate hybrid assemblies via Unicycler (v0.4.8).

Assemblies were annotated using Bakta (v1.9.4; database v5.1, light) [25], and genome comparisons were conducted using blastn and the Mauve progressive alignment plugin [26] for Geneious^®^ (v10.2.6). Circular plasmid visualization was performed using a Python package, pyCirclize (https://github.com/moshi4/pyCirclize/). Synteny maps were produced using pyGenomeViz (https://github.com/moshi4/pyGenomeViz/) and Clinker [27] to align conjugated regions between *E. faecium* donors and transconjugants.

### Identifying conjugation experimental candidates

The ARGs of interest, *vanA*, *tet*(M) and *erm*(B), were the most prevalent across all resistance categories in our dataset. *E. faecium* donors isolates (*n*=3) were identified by screening for a mobile *vanA* gene cluster, *tet*(M) or *erm*(B) located on contigs predicted to represent conjugative plasmids using the MOB-type tool (v3.1.9) [28] (Table 1, part A). Bovine-associated *E. hirae*, a species rarely associated with human infections, carrying *tet*(M), was also selected as a donor (*n*=1; Table 1, part A).

The conjugation recipient candidates of *E. faecium* (*n*=3), *E. faecalis* (*n*=4) and *E. hirae* (*n*=4) were selected based on their lack of overlapping ARGs with the donors and the phenotypic confirmation of susceptibility to vancomycin, doxycycline and erythromycin (Table 1, part B). Bakta annotation was also used to confirm that no CRISPR-Cas genes were present in recipients. Rifampicin and fusidic acid resistance were induced in all recipient strains to serve as markers for selecting recipients or transconjugants in conjugation experiments. Recipient strains were exposed to increasing concentrations of fusidic acid (FA) (5 µg ml^−1^, 10 µg ml^−1^ and 25 µg ml^−1^), followed by gradual exposure to rifampicin (RIF) (5 µg ml^−1^, 10 µg ml^−1^, 50 µg ml^−1^ and 100 µg ml^−1^). The final concentrations of 25 µg ml^−1^ for fusidic acid and 100 µg ml^−1^ for rifampicin were used to select for resistant phenotypes. Phenotypic multidrug resistance in recipients was confirmed by growing isolates on agar plates supplemented with FA and RIF for at least two generations prior to conducting conjugation experiments. The donor and recipient combinations for conjugation experiments are described in Table 2.

**Table 2.** Average conjugation frequencies of *E. faecium* donors containing pVRE0008 with *vanA* gene cluster, pEnt0189_4 with *tet*(M) or NS0794_4 with *erm*(B) and *E. hirae* donor with *tet*(M). The agar-plate mating approach with three replicates was used to estimate average conjugation rates. nt stands for ‘no transfer’, representing failed conjugation.

MGE	Recipient species	Recipient ID	Recipient origin	Conju frequency mean	Conju frequency std.
pVRE0008 with *van*	*E. faecium*	ATCC 51558*	ATCC strain	2.73E-03	1.83E-03
pVRE0008 with *van*	*E. faecium*	NS1659	Clinical	2.26E-04	2.83E-05
pVRE0008 with *van*	*E. faecium*	ES-M-ST001-03MAR15-0067A	Urban wastewater	2.17E-05	1.64E-05
pVRE0008 with *van*	*E. faecalis*	ATCC 47077	ATCC strain	7.41E-08	6.14E-08
pVRE0008 with *van*	*E. faecalis*	ES-C-ST001-01FEB16-0145C	Urban wastewater	1.40E-08	8.34E-09
pVRE0008 with *van*	*E. faecalis*	NS0672	Clinical	6.89E-09	3.16E-09
pVRE0008 with *van*	*E. hirae*	ATCC 9790*	ATCC strain	6.32E-04	2.60E-04
pVRE0008 with *van*	*E. hirae*	ES-V-CB001-26MAY15-0093A*	Feedlot catchbasin	3.67E-06	3.91E-06
pVRE0008 with *van*	*E. hirae*	ES-A-SW-14APR14-Ent0007*	Natural water sources	6.20E-04	4.33E-04
pEnt0189_4 with *tet*(M)	*E. faecium*	NS1659	Clinical	3.78E-04	8.58E-05
pEnt0189_4 with *tet*(M)	*E. faecium*	ES-M-ST001-03MAR15-0067A	Urban wastewater	9.90E-05	1.24E-04
pEnt0189_4 with *tet*(M)	*E. faecalis*	ATCC 47077	ATCC strain	4.40E-08	1.40E-08
pEnt0189_4 with *tet*(M)	*E. faecalis*	ES-C-ST001-01FEB16-0145C	Urban wastewater	nt	nt
pEnt0189_4 with *tet*(M)	*E. faecalis*	NS0672	Clinical	nt	nt
pEnt0189_4 with *tet*(M)	*E. hirae*	ATCC 9790*	ATCC strain	5.84E-04	1.36E-04
pEnt0189_4 with *tet*(M)	*E. hirae*	ES-V-CB001-26MAY15-0093A	Feedlot catchbasin	nt	nt
pEnt0189_4 with *tet*(M)	*E. hirae*	ES-M-ST001-23NOV14-0060A*	Urban wastewater	2.50E-08	3.35E-08
NS0794_4 with *erm*(B)	*E. faecium*	ATCC 51558*	ATCC strain	2.67E-02	2.45E-02
NS0794_4 with *erm*(B)	*E. faecium*	NS1659	Clinical	1.01E-03	3.21E-05
NS0794_4 with *erm*(B)	*E. faecium*	ES-M-ST001-03MAR15-0067A	Urban wastewater	2.71E-05	9.73E-06
NS0794_4 with *erm*(B)	*E. faecalis*	ATCC 47077	ATCC strain	5.89E-07	2.35E-07
NS0794_4 with *erm*(B)	*E. faecalis*	NS0672	Clinical	nt	nt
NS0794_4 with *erm*(B)	*E. faecalis*	BP R37-3	Retail beef	3.27E-07	2.06E-07
NS0794_4 with *erm*(B)	*E. hirae*	ATCC 9790*	ATCC strain	4.17E-03	3.29E-04
NS0794_4 with *erm*(B)	*E. hirae*	ES-V-CB001-26MAY15-0093A	Feedlot catchbasin	9.97E-08	8.64E-08
NS0794_4 with *erm*(B)	*E. hirae*	ES-A-SW-14APR14-Ent0007*	Natural water sources	5.57E-04	1.12E-04
*E. hirae tet*(M)	*E. faecium*	NS1659	Clinical	nt	nt
*E. hirae tet*(M)	*E. faecalis*	NS0672	Clinical	nt	nt
*E. hirae tet*(M)	*E. hirae*	ES-V-CB001-26MAY15-0093A	Feedlot catchbasin	nt	nt

* Transconjugants derived from this recipient were subjected to long-read sequencing.

### Plasmid replicon typing and MGE classification via clustering

Plasmid replicon typing with hybrid assembled genomes of the donor isolates was completed using the web-based version of PlasmidFinder (v2.0.1) [29,30], with the Gram-positive reference database and default settings of 95% minimum identity and 60% minimum coverage. The tool uses a curated database of plasmid replicons to identify plasmids in raw reads, assembled contigs or in whole genomes and to link query plasmids to reference plasmids.

Plasmid type and clusters were predicted using the reference-based MOB-clustering tool of MOB-suite (v3.1.9), based on replicon family, relaxase type, biomarkers and the Mash [31] approximation distance to reference plasmids [28,32]. A reference-free, graph-based Mge-cluster (v1.1.0) approach was used to group the predicted conjugative contigs by the MOB-typer tool of MOB-suite, to generate more detailed distance measures [33]. Mge-cluster was used to analyse the entire input sequence by extracting unitig sequences formed by merging all overlapping reads that could be combined without ambiguity or branching [34]. A perplexity value of 65 was used, and a minimum of 10 sequences was required to define a cluster using Mge-cluster.

### Bacterial growth conditions and conjugation experiments

*E. faecium*, *E. faecalis* and *E. hirae* donor and recipient strains were grown statically in brain heart infusion broth (BHI) (BD Difco) supplemented with vancomycin (32 µg ml^−1^), doxycycline (16 µg ml^−1^) or erythromycin (8 µg ml^−1^) at 37 °C, without CO_2_, for a minimum of 14 h. Antibiotics were included at breakpoint concentrations according to the Clinical and Laboratory Standards Institute documents M02-A1284 and M100-S24. Overnight grown bacterial donor and recipient cultures were subcultured in fresh BHI broth without antibiotics to achieve an OD600 of 0.1 and harvested at the exponential growth phase of 0.7-0.8 by centrifuging at 9,000 ***g*** for 2.5 min. The bacterial pellets were resuspended in 500 µl of the subculture supernatant. A portion of each resuspension was used to determine cell density via serial tenfold dilutions in PBS, with 10⁻⁶ dilutions plated to verify that equal numbers of donor and recipient cells were used for mating. Concentrated donor and recipient cultures were then mixed 1:1 (250 µl each), yielding 500 µl of mixed culture for mating. The mixture was plated on BHI agar as five 100 µl spots (i.e. mating spots) and statically incubated overnight at 37 °C.

**Fig. 1.**
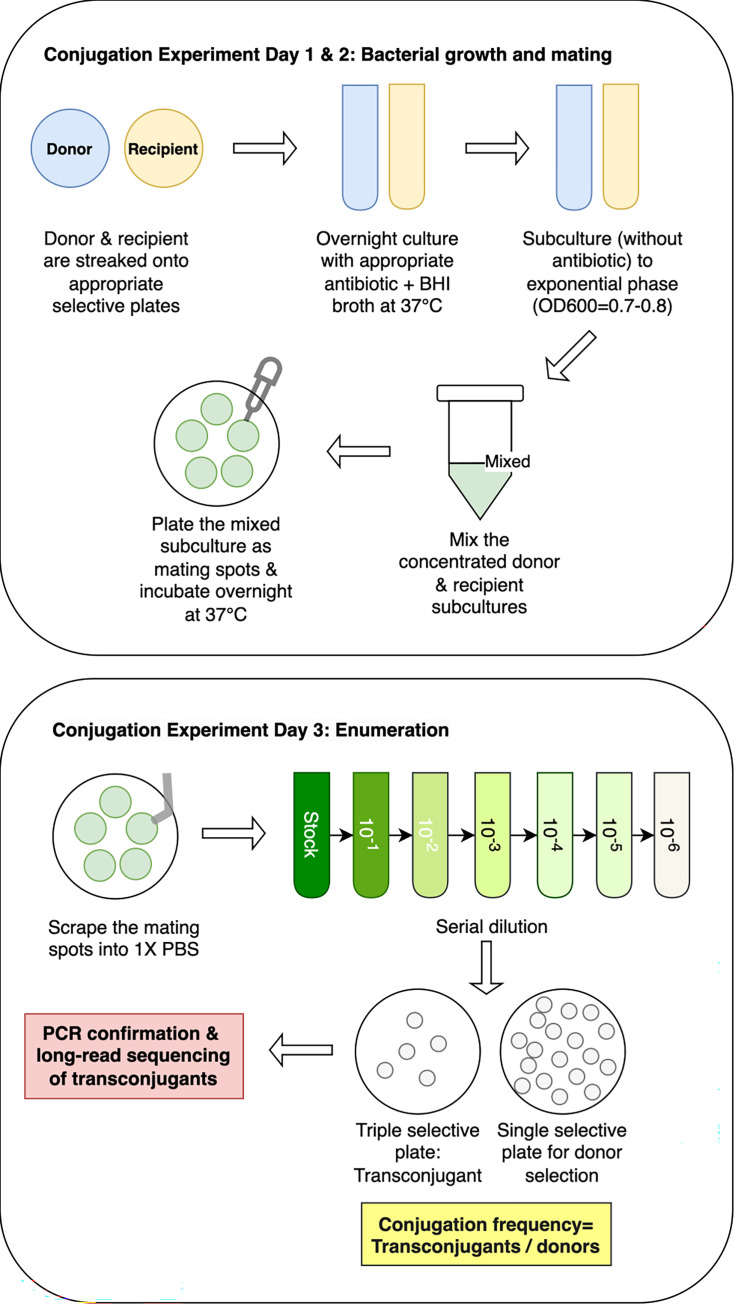
A summary visualization of the conjugation experiment, with donors and recipients presented in Table 1. The donor-recipient combinations used in the conjugation experiments are shown in Table 2.

Following overnight incubation, mating spots were scraped into 1 ml of 1X PBS, and serial dilutions (diluted to 10^−7^) were plated on selective agar plates. For all mating combinations, BHI agar supplemented with antibiotics was used to select for donor (vancomycin, doxycycline or erythromycin), recipient (rifampin and fusidic acid) and transconjugant (rifampin, fusidic acid and the antibiotic that the donor was resistant to) populations. Transconjugants were incubated for up to 48 h at 37 °C, while the growth of donor and recipient cells was assessed after 24 h at 37 °C. Plates containing 30-300 colonies were used to calculate colony-forming units (c.f.u.) per millilitre, except for transconjugants, for which fewer than 30 colonies were occasionally recovered from undiluted mating spot suspensions. Conjugation frequency was calculated as the number of transconjugant c.f.u. divided by the number of donor c.f.u. Transconjugants were streaked on triple-selective plates for two generations after conjugation to confirm the stability of the AMR phenotype. Fig. 1 presents a summary visualization of the conjugation experiment.

### Confirmation of transconjugants

To confirm the transfer of ARG-carrying MGEs from donors to recipients, PCR assays were performed using *vanH*, *tet*(M) or *erm*(B) primers. Whole-cell templates of transconjugants were prepared by suspending a single bacterial colony in 50 µl of sterile TE buffer (pH 8.0), followed by heat lysis at 95 °C for 15 min. Amplified PCR products were separated by electrophoresis on a 2.0% agarose gel and visualized by staining with GelRed^®^ Nucleic Acid Gel Stain (Biotium, Fremont, USA). High-molecular-weight genomic DNA was extracted from selected transconjugants using the Genomic-tip 20 G^−1^ kit (QIAGEN, Hilden, Germany) and subjected to whole-genome sequencing. Nanopore libraries were prepared using the Native Barcoding Kit 96 V14 (Oxford Nanopore, Oxford, UK). Briefly, high-molecular-weight genomic DNA from each bacterial sample was repaired and end-prepared, followed by ligation of a unique barcode from the kit to the DNA ends. After barcoding, all samples were pooled, and sequencing adapters were ligated to the combined library. The pooled, barcoded library was then loaded onto an R10.4.1 flow cell, and sequencing was performed using the MinKNOW software. Reads were demultiplexed and assigned to their respective samples using the barcode sequences. The list of transconjugants is indicated in Table 2.

## Results

### Identification of MGEs carrying antibiotic resistance genes

**Fig. 2.**
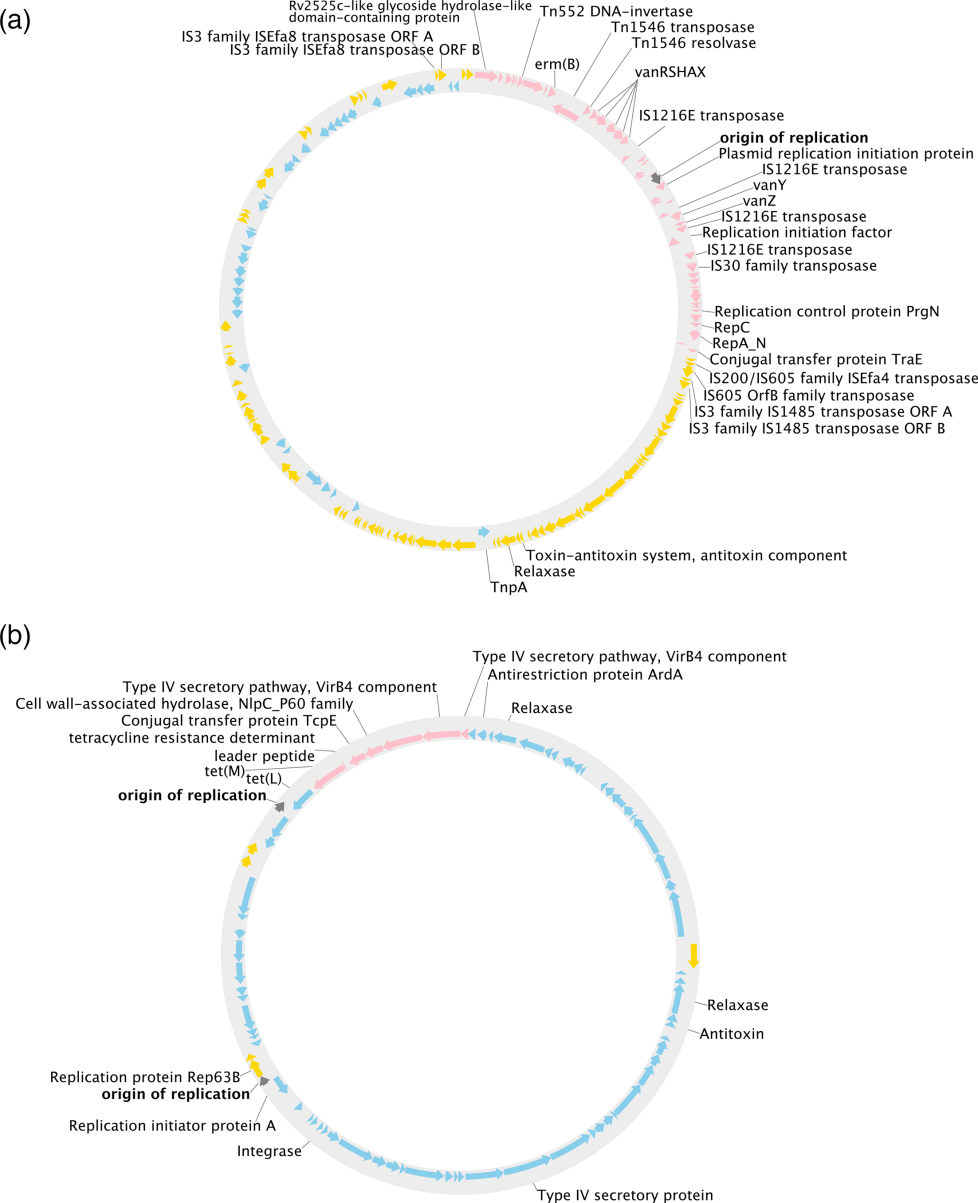
Resolved plasmid structures. (**a**) pVRE0008 (232,902 bp) was identified from the donor *E. faecium* isolate VRE0008. The pink arrows containing Tn*1546* transposase, Tn*1546* resolvase and *vanRSHAX* genes transferred consistently to recipients as confirmed by long-read assemblies of *E. faecium* ATCC, *E. hirae* ATCC, *E. hirae* 0093 and *E. hirae* Ent0007 transconjugants (refer to Fig. 6 for a complete annotation of the region). (b) pEnt0189_4 (73,578 bp) was identified in *E. faecium* isolate Ent0189. The region homologous to the canonical Tn*916* ICE (GenBank: U09422.1; 18,032 bp), including the *tet*(M), conjugal transfer protein, cell wall-associated hydrolase of NlpC/P60 family, and the VirB4 components of the type IV secretory pathway, is represented in pink.

*E. faecium* donor VRE0008 contained a 232,902 bp plasmid, designated pVRE0008, as predicted by MOB-suite and confirmed through sequencing of transconjugants (Fig. 2). Several essential elements of the conjugation machinery, including the type IV secretory and conjugal transfer genes, were observed in pVRE0008, along with a variety of transposase genes from different insertion sequence families (Fig. 2a). pVRE0008 carried the vancomycin resistance-*vanA* gene cluster encoding the VanS/VanR two-component system that senses extracellular glycopeptides, and VanHAX, which alters peptidoglycan precursors to evade the inhibition of cell wall biosynthesis by this antibiotic [35]. The *vanA* gene cluster in pVRE0008 was associated with Tn*1546*, as indicated by the presence of Tn*1546* transposase and resolvase genes directly upstream of the cluster. A 38-bp inverted repeat (IR) sequence (GGGGTAGCGTCAGGAAAATGCGGATTTACAACGCTAAG), identical to the left IR of Tn*1546* in *E. faecium* BM4147 [36], was also located upstream and adjacent to the transposase gene; however, no corresponding downstream IR was detected. Additionally, the *vanA* cluster was followed by an IS1216E transposase gene, suggesting the formation of an IS1216-bound ‘pseudo-compound transposon’ (Fig. 2a [37]). pVRE0008 also carried the *erm*(B) gene, which confers high-level resistance to macrolides, lincosamides and streptogramin B (MLS_B_ phenotype) through translation attenuation [38].

A circularized contig (73,578 bp) in the donor *E. faecium* Ent0189 was designated as plasmid pEnt0189_4 (Fig. 2b). This plasmid carried *tet*(M) and *tet*(L), which confer tetracycline resistance through ribosomal protection and antibiotic efflux, respectively [39,40]. To investigate whether *tet*(M) was associated with Tn*916*, regions surrounding *tet*(M) in pEnt0189_4 were compared with the canonical Tn*916* sequence (GenBank: U09422.1; 18,032 bp) in the ICEberg 2.0 database [41], revealing a pairwise identity of 84.2% (highlighted in Fig. 2b). However, pEnt0189_4 lacked the transposase and excisionase genes of Tn*916*.

A contig from *E. faecium* NS0794 (140,080 bp), carrying *erm*(B), was predicted as conjugative by MOB-suite but lacked the origin of replication needed to form a plasmid and was designated NS0794_4.

### Plasmid classification with replicon typing

**Fig. 3.**
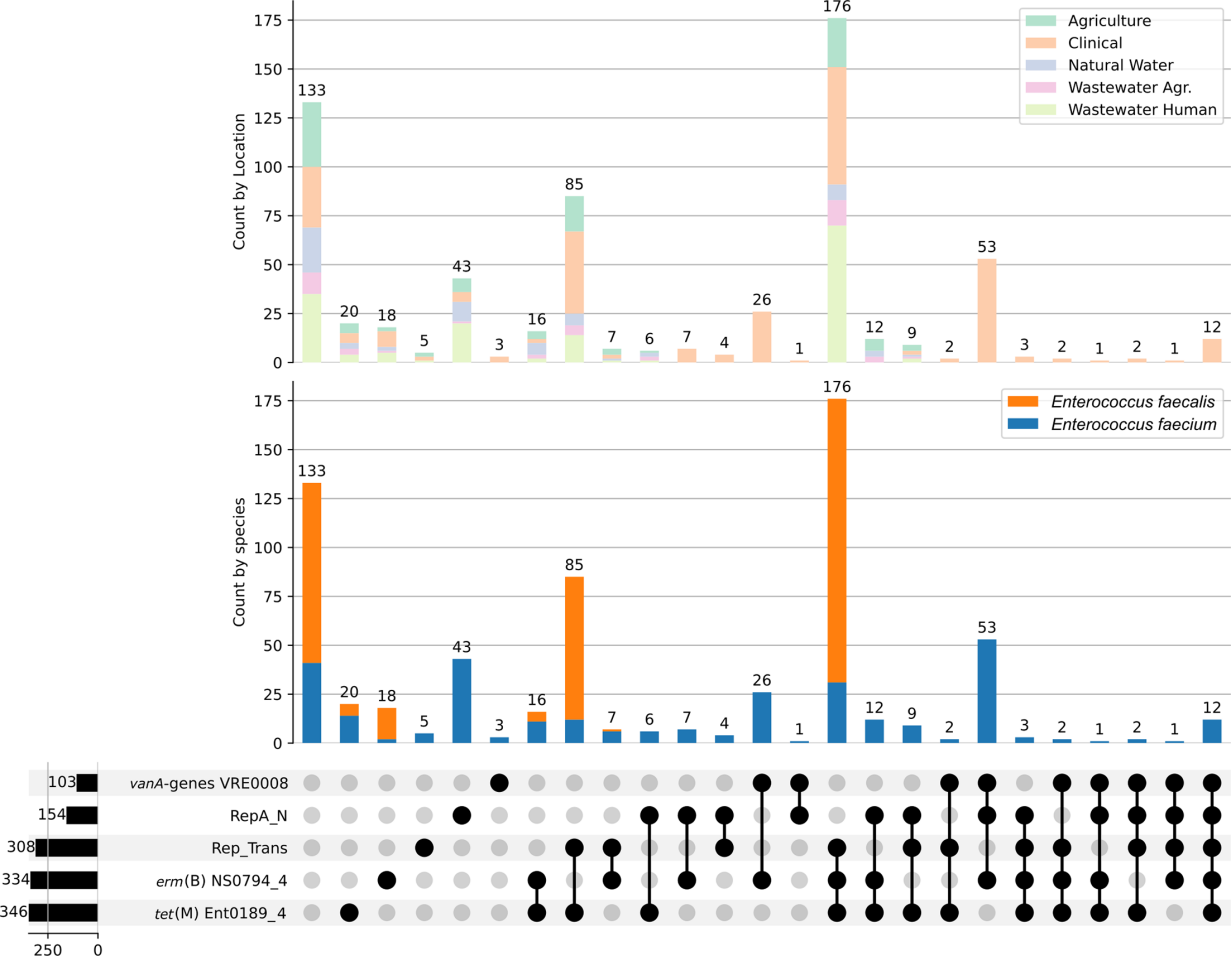
Intersection plot representing the co-occurrence of replicon types and antimicrobial resistance genes in 309 *E. faecium* and 338 *E. faecalis* isolates across the One Health continuum. Of the 103 isolates with the *vanA* gene cluster, 70 contained the RepA_N-type replicon; of the 334 isolates with the *erm*(B) gene, 89 contained the RepA_N-type replicon; and of the 346 isolates with the *tet*(M) gene, 291 contained the Rep_Trans-type replicon.

The replication module of pVRE0008 was identical to the RepA_N-type replicon identified as ‘repUS15’ by PlasmidFinder (reference accession CP003586, DO3 plasmid 3). The RepA_N-type replicons are widely distributed among low G+C Gram-positive bacteria, but plasmids harbouring RepA_N are typically of narrow host range and unable to replicate outside their native hosts [42]. Out of the 647 genomes, only 154 contained the RepA_N-type replicon with a minimum percent identity of 95.5% (Fig. 3). Less than half of the 154 *E. faecium* isolates with the RepA_N replicon (*n*=70) carried the *vanA* gene cluster and those that did were of clinical origin (i.e. vancomycin-resistant enterococci, VREs) (Fig. 3). These results demonstrate that not all RepA_N-type plasmids were associated with the *vanA* gene cluster. The replication module of pEnt0189_4 was identical to the Rep_Trans-type replicon identified as ‘repUS43’ by PlasmidFinder (reference accession CP003584, DO plasmid 1). Out of the 647 genomes, 308 contained the Rep_Trans-type replicon with a minimum percent identity of 89.6% (Fig. 3). A total of 291 (∼94.5%) of these carried *tet*(M) and were predominantly *E. faecalis* (*n*=218) of clinical or human wastewater origin (Fig. 3). Our results show that most *tet*(M) genes (total *n*=346) in our dataset were associated with Rep_Trans-type plasmids.

**Fig. 4.**
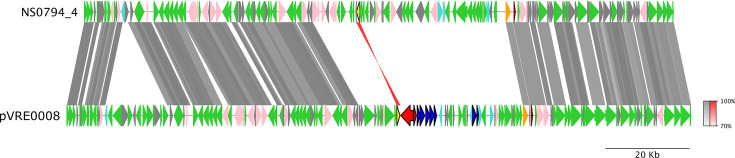
Synteny comparison between *E. faecium* NS0794_4 and pVRE0008. Grey blocks mark homologous regions, and the red block marks reverse-complement homology. Darker shades indicate higher sequence similarity, as shown by the scale bar. Arrows mark CDS and their transcriptional direction. Turquoise arrows highlight IS1216E transposase genes, while pink indicates other transposase genes. The yellow arrow marks the 23S rRNA (adenine[2058]-N6)-methyltransferase Erm(B) CDS, red arrows mark the Tn*1546* transposase and Tn*1546* resolvase genes and blue arrows mark the VanRSHAX and VanYZ CDS. The orange arrow highlights the RepA_N-type protein, and brown indicates the relaxase gene shared between the two sequences.

The NS0794_4 replication module also perfectly matched the ‘repUS15’ RepA_N-type replicon. However, unlike VRE008, * E. faecium* NS0794 did not contain vancomycin ARGs. Approximately half (*n*=89) of the total RepA_N-type replicon-harbouring isolates (*n*=154) carried *erm*(B) and were predominantly *E. faecium* of clinical origin (Fig. 3). Sixty-seven of the 89 isolates with RepA_N-type replicon and *erm*(B) also contained the *vanA* gene cluster (Fig. 3), implying transfer of multidrug resistance by RepA_N-type plasmids. When comparing pVRE0008 and NS0794_4, several shared regions were observed (Fig. 4). However, a region surrounding the Tn*1546*-type *vanA* gene cluster and a 10-kb area containing genes associated with carbohydrate metabolism were absent in NS0794_4 (Fig. 4).

### Plasmid classification with clustering

Both pVRE0008 and NS0794_4 are members of the MOB-suite primary cluster AC733. In total, 249 contigs from 144 genomes were assigned to the AC733 plasmid cluster, with 79 genomes containing more than 1 contig belonging to this cluster. When comparing the replicon-typing and plasmid cluster prediction results using short-read assemblies, there was no overlap between contigs containing the RepA_N-type replicon and those within the AC733 plasmid cluster. Of the 85 genomes identified as either RepA_N plasmid types or associated with the AC733 plasmid cluster, 94% were clinical *E. faecium*.

The pEnt0189_4 plasmid was assigned to the primary MOB-cluster AB756, which included a total of 1197 contigs from 445 genomes, with 300 genomes containing more than 1 contig from this cluster. Forty-three contigs contained the Rep_Trans-type replicon and were classified as belonging to the AB756 plasmid cluster. Of the 647 genomes, 239 had contig(s) identified as either Rep_Trans types or associated with the AB756 plasmid cluster. Of these, 151 were *E. faecalis*, primarily of human wastewater and clinical origin, while 88 were *E. faecium* from clinical, agricultural, wastewater and natural water sources.

**Fig. 5.**
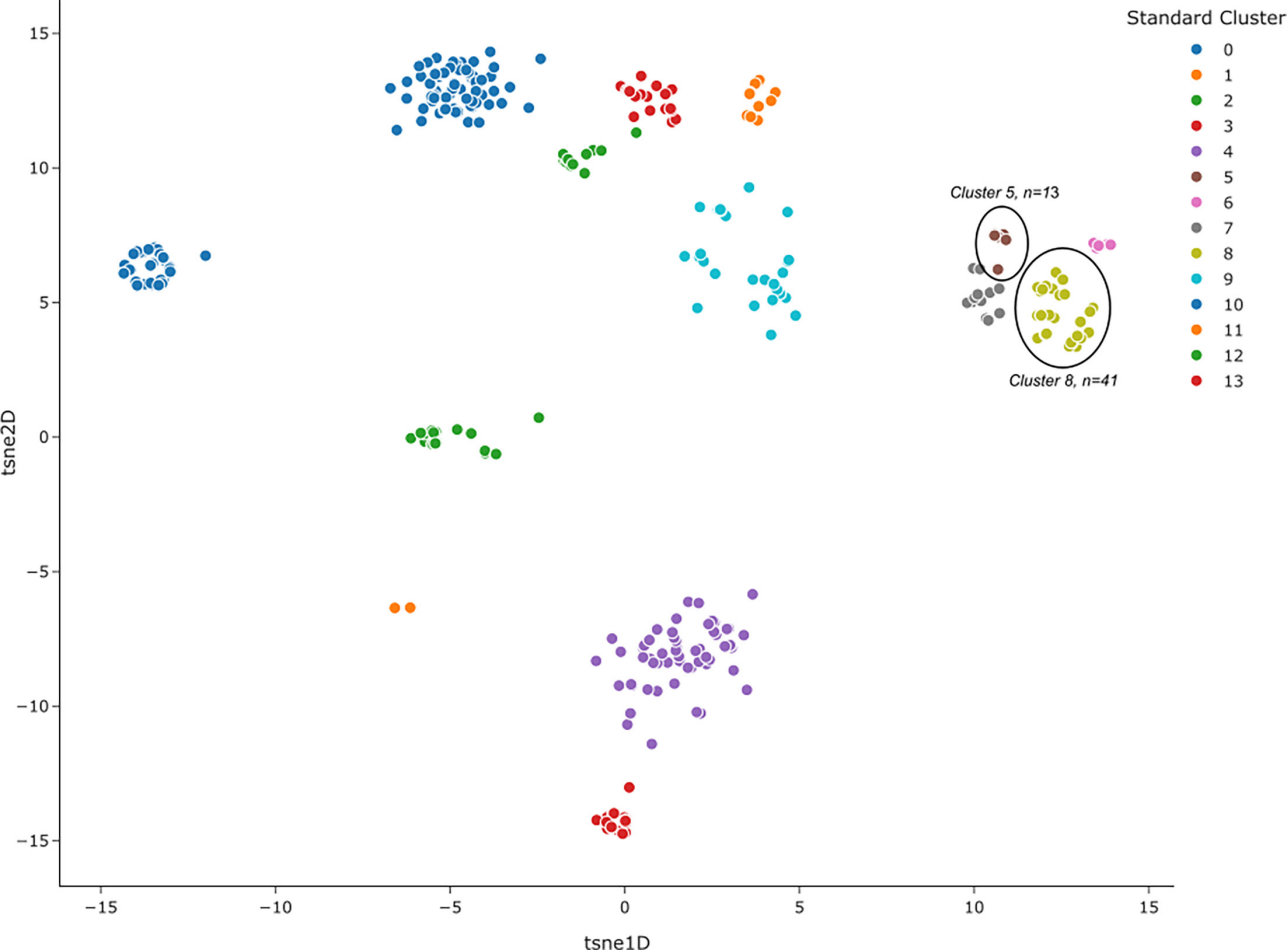
Putative enterococci plasmid clustering via Mge-cluster (v1.1.0). Each point corresponds to a potential plasmid sequence (i.e. contigs defined as conjugative via MOB-suite). pVRE0008 and NS0794_4 were members of cluster 5 (brown, *n*=13), which also clustered together in replicon-typing and reference-based clustering methods. pEnt0189_4 was assigned to cluster 8 (sage yellow, *n*=41).

Using MGE-Cluster, pVRE0008 and NS0794_4 were grouped into standard cluster 5 (Fig. 5), while pEnt0189_4 was assigned to standard cluster 8 (Fig. 5). Most members of standard cluster 8 (*n*=41) lacked *tet*M or *tet*L. Compared to the replicon typing and reference-based clustering, which also classified pVRE0008 and NS0794_4 together, reference-free clustering formed a much smaller cluster composed of 13 isolates.

Of the 13 isolates in standard cluster 5, all except 2 possessed *erm*(B), while 4 isolates did not contain the *vanA* gene cluster, but 1 of these (HC_NS1332) did contain the *vanHAX* cluster. All isolates in standard cluster 5 exhibited shared plasmid regions with pVRE0008 and NS0794_4, including the RepA_N replicon, conjugal transfer protein TraE, components of the type IV secretion system, relaxase and the antitoxin component of the toxin–antitoxin system (Fig. S1, available in the online Supplementary Material).

### Conjugation frequencies of MGEs containing ARGs and the structure of transferred MGEs

The *E. faecium* donor with pVRE0008 conjugated with all recipients at varied rates, whereas conjugation of *E. faecium* pEnt0189_4 and NS0794_4 donors only occurred with select recipients (Table 2). With *E. faecium* pVRE0008 as a donor, conjugation frequencies were highest with *E. faecium* recipients (10^−3^ to 10^−5^ transconjugants per donor, T/D), followed by *E. hirae* (10^−4^ to 10^−6^ T/D) and *E. faecalis* (10^−8^ to 10^−9^ T/D).

Similarly, with *E. faecium* pEnt0189_4 as a donor, conjugation frequencies were the highest with *E. faecium* recipients (10^−4^ to 10^−5^ T/D), followed by *E. hirae* (10^−4^ to 10^−8^ T/D), with one of these isolates failing to conjugate. Conjugation with *E. faecalis* recipients was the least frequent, with only one successful event (10^−8^ T/D).

With *E. faecium* NS0794_4 as a donor, conjugation frequencies ranged between 10^−2^ and 10[Aff aff2]^−6^ T/D in *E. faecium* recipients, 10^−4^ and 10^−8^ T/D in *E. hirae* recipients and 10^−7^ T/D in *E. faecalis* recipients, with one failed event. The *E. faecium* pEnt0189_4 donor appeared to have the lowest conjugation rate, while *E. faecalis* clinical isolate NS0672 was the least receptive to conjugation.

**Fig. 6.**
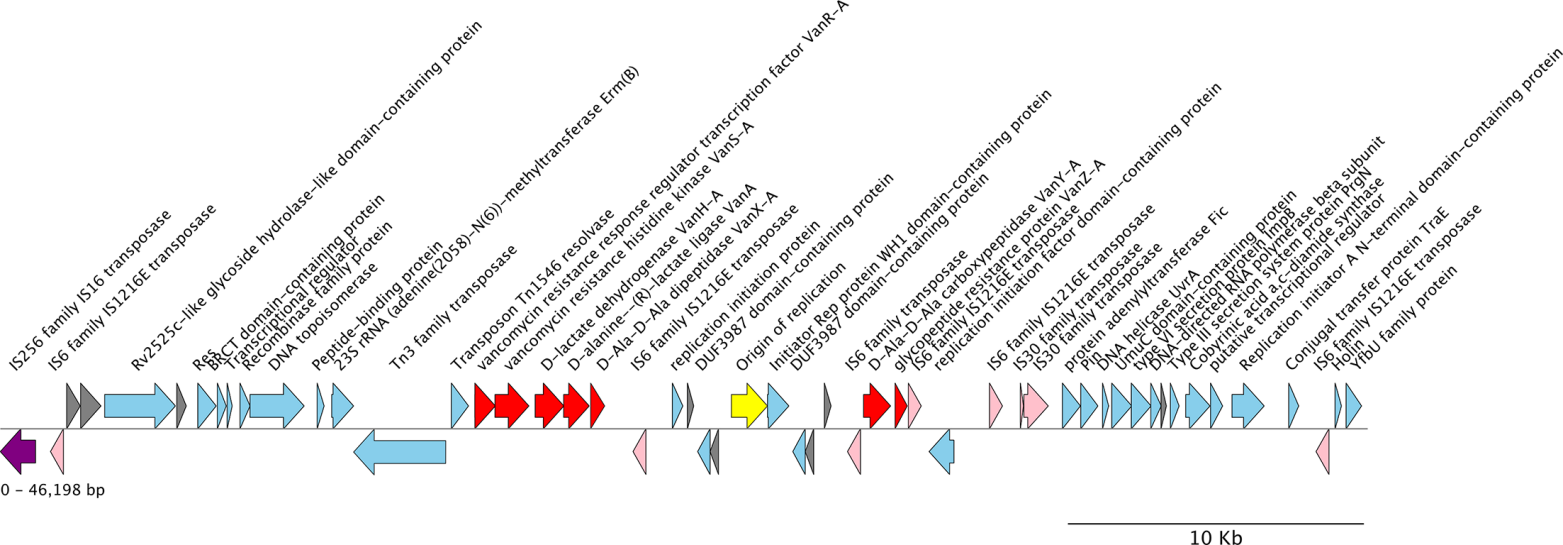
Long-read assembly of VRE0008 resolved a 46,198 bp contig encompassing the *vanA* gene cluster. Red arrows indicate genes contributing to vancomycin resistance, grey arrows represent hypothetical protein genes, pink arrows indicate IS transposase genes, and the purple arrow denotes the IS256 family transposase gene IS16. All other genes are shown in blue. This module is presented as an independent contig in transconjugants (*E. hirae* ATCC TC, *E. faecium* ATCC TC, and *E. hirae* Ent0007 TC), but is integrated into a larger plasmid flanked by IS16 transposase genes in *E. hirae* 0093 TC.

Long-read assemblies (*n*=4) confirmed that the *vanA* gene cluster was consistently transferred via conjugation as part of a 46,198 bp unit (Fig. 6). The transconjugant, *E. hirae* ES-V-CB00126MAY15-0093A, was the exception (*E. hirae* 0093 TC), as when *E. faecium* pVRE0008 was the donor, the whole plasmid (232,902 bp) was transferred. For *E. hirae* ATCC 9790 (*E. hirae* ATCC TC), *E. faecium* ATCC 51558 (*E. faecium* ATCC TC) and *E. hirae* ES-A-SW-14APR14-Ent0007 (*E. hirae* Ent0007 TC) transconjugants, only the 46-kb surrounding *vanA* gene cluster was transferred.

To investigate the structure of this 46-kb segment in the transconjugant long-read assemblies, we examined the long-read assembly of the donor genome. Unlike the hybrid assembly, the long-read assembly of VRE0008 did not resolve a complete plasmid and instead yielded a separate 46,198 bp contig that mapped to a section of a plasmid of *E. hirae* 0093 TC (Fig. S2). In *E. hirae* 0093 TC, the region was flanked by IS16 transposase genes, suggesting its potential as a ‘pseudo-compound transposon’ (Fig. S2). However, because the transconjugants were assembled using long reads alone and contained a high density of ISs, assemblies may be incomplete, making it difficult to determine whether the 46-kb segment is part of a larger replicon (chromosome or plasmid) in other transconjugants.

Long-read sequencing of the transconjugants (*n*=3) with the NS0794 donor revealed contigs that aligned with the entire sequence of NS0794_4. This suggests that the entire putative MGE was horizontally transferred in these isolates. Comparison of long-read assemblies of transconjugants (*n*=2) with the Ent0189 donor also confirmed the transfer of the entire pEnt0189_4 plasmid (73,578 bp, Fig. 2b). Transfer of the whole plasmid makes monitoring and tracing HGT easier than modular transfer of MGEs.

Finally, the *E. hirae* donor with *tet*(M) and *tet*(L) genes localized to the Rep_Trans-type plasmid, similar to pEnt0189_4, did not conjugate (Table 2).

## Discussion

*Enterococcus* species have highly plastic genomes that allow them to quickly adapt to and survive in dynamic environments [11]. These opportunistic pathogens can accumulate ARGs and serve as reservoirs of AMR dissemination [43]. In this investigation, we described and assessed the abundance and dissemination potential of MGEs bearing ARGs across three enterococci species, and the horizontally transferred MGEs were characterized.

The RepA_N-type conjugative plasmids contained the *vanA* vancomycin resistance gene cluster, commonly associated with Tn*1546* (Fig. 2a), and the erythromycin-resistance gene *erm*(B) (Fig. 4). Previous studies have demonstrated a common association of the *vanA* gene cluster with RepA_N plasmids in various *E. faecium* strains linked to hospital outbreaks, pigs and non-hospitalized populations [12,21]. Our RepA_N replicon was exclusively found in *E. faecium*, being identified in ~25% of genomes, but was not always associated with the *vanA* gene cluster. Observed differences between pVRE0008 and NS0794_4 are likely due to the reshuffling of their replication, partition and conjugative components, as has been observed in RepA_N type plasmids by others, a characteristic that may aid in host and niche adaptation [42]. Despite these differences, the reference-based and reference-free clustering methods consistently grouped pVRE0008 with NS0794_4, with the reference-free method generally yielding considerably smaller clusters. This disparity may reflect differences in the scope of comparison, as the reference-free method evaluates the entire sequence of contigs predicted to be MGEs, which will capture the variation in mosaic RepA_N-type plasmids and generate smaller clusters. These findings demonstrate that MGE classification based on specific genes as representatives of a complete structure may fail to provide a comprehensive understanding of the gene repertoire for highly mosaic structures.

The Rep_Trans-like conjugative plasmid pEnt0189_4 carried the doxycycline resistance-conferring *tet*(M) gene. The Rep_Trans replicon co-occurred with *tet*(M) in both *E. faecium* and *E. faecalis* isolates, although it was more common in *E. faecalis*. By reference-based and reference-free methods, pEnt0189_4 was assigned to clusters that were considerably larger than pVRE0008 and NS0794_4. These results collectively suggest that the Rep_Trans-type plasmid is a more abundant ARG-carrying MGE in our dataset, distributed across *E. faecium* and *E. faecalis*, as compared to the RepA_N-type plasmid, which was exclusively hosted by clinical isolates of *E. faecium*. Plasmids of the Rep_Trans group are found in various enterococci and staphylococci species and have been previously shown to carry tetracycline or chloramphenicol ARGs [44,45]. Despite the broader prevalence of the Rep_Trans-type plasmid, its observed conjugation rates were not as high as those of the RepA_N-type plasmids. Abundance does not necessarily indicate a higher transfer rate and may instead reflect more stable plasmid carriage, possibly due to a lower fitness cost to the host [46,48].

ARGs residing on MGEs theoretically pose a greater risk than those integrated into the chromosome as they are more readily transferred across bacterial species that are potentially harmful to humans and livestock [49,52]. In our study, vancomycin-resistance genes from an *E. faecium* donor were successfully transferred to all recipients of all three species. Our findings align with previous studies demonstrating consistent interspecies conjugation between *E. faecium* donors to *E. faecalis* recipients, of clinical and agricultural origins, with the *vanA*-gene associated with Tn*1546* harboured on conjugative plasmids [53,55]. Studies showed that the elements mediating enterococci *vanA*-type resistance show remarkable diversity in structure due to insertion sequences like IS1216V or by fusing with other plasmids to enhance mobility [56,58]. Different transposition patterns of *vanA*-carrying MGEs were observed in this study, including cases where either only Tn*1546* was transferred or the entire plasmid harbouring the transposon was mobilized. These findings expand our understanding of the dynamic structures that facilitate AMR dissemination.

It has long been theorized that general properties of plasmids, such as size and gene expression burden, impose fitness costs on the host due to the bioenergetic demands of plasmid maintenance [46]. However, increasing evidence suggests that conflicts between MGEs and resident chromosomal genes may play a larger role in limiting plasmid transfer and persistence [59]. Because single compensatory mutations can often fully ameliorate these fitness costs, specific host-plasmid genetic interactions have been proposed as key barriers to plasmid establishment in new hosts. This is exemplified in *Pseudomonas fluorescens*, where a single mutation in a putative gene alleviated the disruption associated with plasmid carriage [59].

These findings may help explain the efficient, modular transfer of the smaller *vanA*-carrying unit, which exhibited the highest conjugation rate among all MGEs tested. If this smaller unit functions as an independent MGE, it may generate fewer genetic conflicts with host resident genes, thereby facilitating more efficient transfer. Alternatively, its apparent independence may reflect assembly limitations. The unit could be integrated into the recipient chromosome, but the high density of ISs flanking the *vanA* cluster may hinder complete assembly in transconjugant genomes. This limitation is expected to be addressed in future studies as sequencing accuracy continues to improve.

Previous studies, along with our findings, reinforce the extensive dissemination strategies employed by enterococci to spread *vanA* through variants of Tn*1546* underscoring the need to develop mitigation measures targeting MGE-mediated HGT that drives AMR [60, 61]. The vectors (i.e. MGEs) that drive ARG spread should be considered an essential therapeutic target, in addition to the hosts carrying them. Using conjugation-inhibiting compounds alongside antibiotics may help hinder resistance spread, such as engineered intracellular antibodies that inhibit relaxase activity in recipient cells [62,64].

Most frequently isolated from cattle, *E. hirae* demonstrated a notable possibility for niche expansion by receiving different ARGs from other enterococci species [1,65]. This is particularly important from a One Health perspective, as the ability of agriculturally prevalent *E. hirae* to acquire and maintain ARG-carrying MGEs could facilitate unexpected transmission pathways between agricultural and clinical environments [66,67]. Our study demonstrated successful conjugative acquisition of MGEs carrying ARGs from diverse sources, which suggests that *E. hirae* can incorporate resistance traits that may enhance survival in environments typically dominated by *E. faecium* and *E. faecalis*. In particular, the bovine-derived isolate *E. hirae* (0093A) was the only recipient to fully retain a RepA_N-type plasmid that carried the *vanA* gene cluster. This finding suggests that strain- or lineage-specific factors may influence plasmid stability, indicating that *E. hirae* could serve as a suitable host for AMR-carrying MGEs and contribute to disseminating ARGs across ecological niches.

Donors containing either *tet*(M) or *erm*(B) did not conjugate in all matings, but when conjugation was successful, the hosts acquired complete plasmids, unlike the modular movement demonstrated by *vanA*-carrying pVRE0008. Typically, *tet*(M) is an accessory cargo gene carried by Tn*916* [68,69]. In our study, most components of the canonical Tn*916* were mapped to pEnt0189_4, except for transposase (*tnp*) and excisionase (*xis*) genes. The first step in conjugative transposition of Tn*916* involves excision from the donor DNA, followed by transposon circularization and transfer [70]. The *tnp* and *xis* genes enhance the frequency of excision and transposition [71]. Without these mechanisms, Tn*916*’s ability to excise from its original location was likely impaired, limiting its independent mobilization. However, previous findings have shown that excision of Tn*916* can lead to host cell growth arrest, suggesting that the excision process itself may impose a fitness cost on the host [72]. We postulate that mechanisms enhancing transfer rates but imposing greater stress on the host may have been ameliorated through compensatory mutations, favouring lower fitness costs and increased plasmid or transposon stability. This aligns with the observed prevalence of *tet*(M)-carrying Rep_Trans-type plasmids in our dataset, as even in the presence of *tnp* and *xis* genes, HGT with the *E. hirae tet*(M) donor did not occur.

Failure to conjugate may result from host-defence mechanisms that inhibit HGT, like the restriction modification (RM) system that distinguishes self from foreign DNA by DNA methylation [73,75]. However, despite pEnt0189_4 encoding the antirestriction protein ArdA, which enables MGE to evade the host RM system, conjugation failed, suggesting that additional factors may be involved (Fig. 2) [76]. The failed intra- and interspecies transfer of *tet*(M) from bovine *E. hirae* suggests that the MGE may carry highly conflicting genes for the host that, along with other host defence mechanisms, impede HGT.

Our study highlights the transmission potential of ARGs in *E. faecium*, *E. faecalis* and *E. hirae*, the boundaries of mosaic MGEs and the potential niche expansion of MGE-mediated ARGs among enterococci. Higher conjugation rates did not necessarily lead to broader dissemination of MGEs in enterococci, demonstrating that predicting dissemination requires understanding both transfer rate (i.e. acquisition cost) and stability (i.e. fitness cost) [48,77, 78]. Our study’s limitation includes the fact that we only observed mating success for two generations following the conjugation experiment and only under selection pressure. Continued monitoring of plasmid persistence across multiple generations (e.g. 50–100 generations) in the absence of selection pressure could have provided insights about the maintenance success of MGEs. Additionally, our analyses were based on a limited number of isolates, and potential conjugation rates with other *Enterococcus* species were not explored, which may also influence transfer dynamics among enterococci occupying different ecological niches. Research should prioritize the comprehensive characterization of MGEs and the systematic, extensive functional testing of their transferability, particularly for those carrying diverse combinations of ARGs. Failed conjugation events should be investigated for the genetic content causing conflicts between incoming MGEs and the host genome. These efforts will provide deeper insights into the architecture of MGEs, conjugation dynamics and their role in disseminating resistance, further strengthening our ability to monitor, control and effectively mitigate the spread of AMR.

## Supplementary material

10.1099/mic.0.001720 Supplementary Material 1.

## Abbreviations

ARGs: antimicrobial resistance genes
BHI: brain heart infusion broth
FA: fusidic acid
HGT: horizontal gene transfer
ICEs: integrative and conjugative elements
IR: inverted repeat
MGEs: mobile genetic elements
ML: machine learning
RIF: rifampicin
RM: restriction modification
T/D: transconjugants per donor
TEs: transposable elements
VREs: vancomycin-resistant enterococci
