# Supplementary material for: Investigating the mobility and host range of mobile genetic elements harbouring antimicrobial resistance genes in enterococci
Source: Microbiology (Reading). 2026 Jul 2;172(7):001720. doi: 10.1099/mic.0.001720 (PMC13339827; doi:10.1099/mic.0.001720)
Supplement: Supplementary Material 1. [file mic-172-01720-s001.pdf]

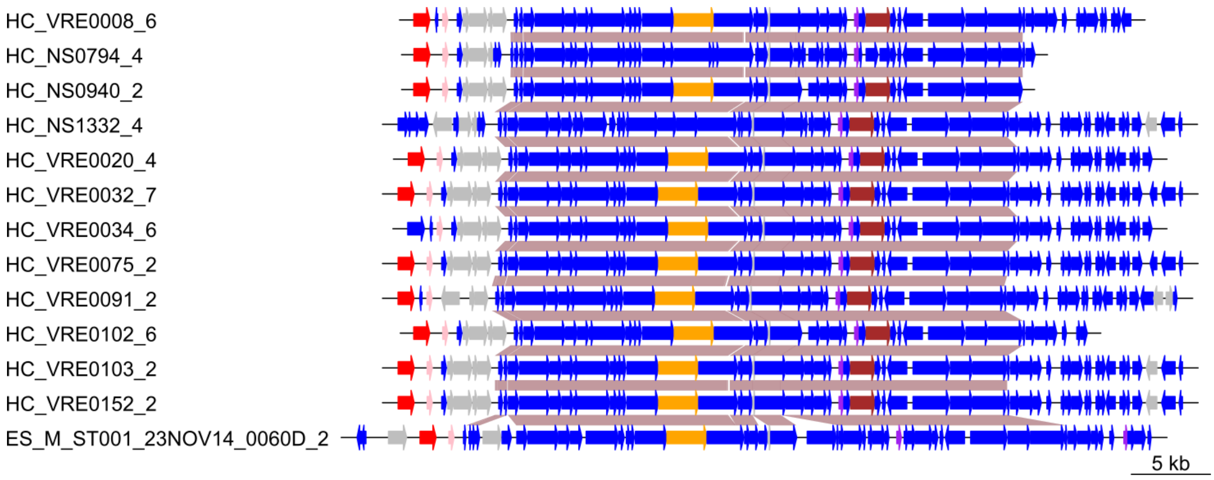

Supplementary Figure 1. Comparison of isolates from standard cluster 5 established via Mge-cluster (v1.1.0), showing aligned regions with highlighted coding sequences: RepA_N-type replicon (red), conjugal transfer protein TraE (pink), type IV secretory system components (orange), relaxase (brown), and the antitoxin component of a toxin-antitoxin system (purple).

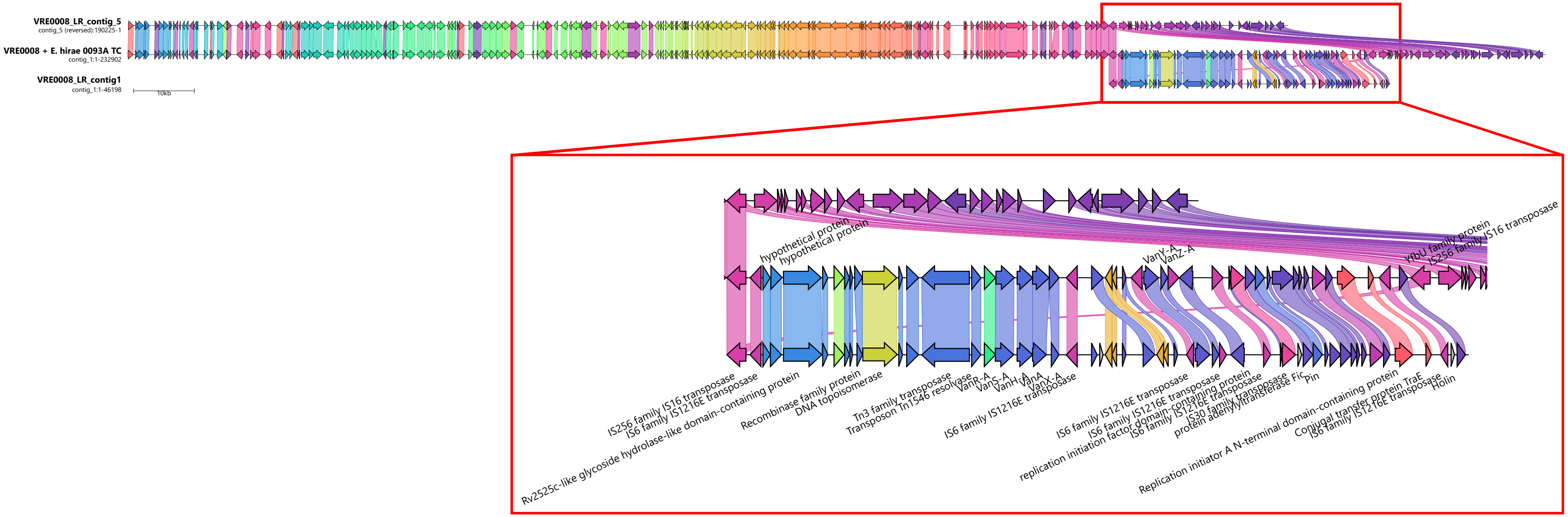

Supplementary Figure 2. Synteny map of coding sequences comparing a putative plasmid from the VRE0008 + *Enterococcus hirae* 0093A transconjugant with two contigs from the VRE0008 long-read (LR) assembly. Arrows indicate predicted genes, and blocks indicate synteny between the sequences, and both are coloured arbitrarily. The unmatched region between the transconjugant putative plasmid and VRE0008_LR_contig1 likely reflects a sequencing artifact. Full annotation of the zoomed region is provided in Figure 6.
